# Role of the Cerebellum in Adaptation to Delayed Action Effects

**DOI:** 10.1016/j.cub.2017.06.074

**Published:** 2017-08-21

**Authors:** Liyu Cao, Domenica Veniero, Gregor Thut, Joachim Gross

**Affiliations:** 1 School of Psychology, University of Glasgow, Glasgow G12 8QB, UK; 2 Institute of Neuroscience and Psychology, University of Glasgow, Glasgow G12 8QB, UK; 3 Department of Psychology (III), University of Würzburg, 97070 Würzburg, Germany

**Keywords:** cerebellum, forward model, MEG, TMS, sensory attenuation

## Abstract

Actions are typically associated with sensory consequences. For example, knocking at a door results in predictable sounds. These self-initiated sensory stimuli are known to elicit smaller cortical responses compared to passively presented stimuli, e.g., early auditory evoked magnetic fields known as M100 and M200 components are attenuated. Current models implicate the cerebellum in the prediction of the sensory consequences of our actions. However, causal evidence is largely missing. In this study, we introduced a constant delay (of 100 ms) between actions and action-associated sounds, and we recorded magnetoencephalography (MEG) data as participants adapted to the delay. We found an increase in the attenuation of the M100 component over time for self-generated sounds, which indicates cortical adaptation to the introduced delay. In contrast, no change in M200 attenuation was found. Interestingly, disrupting cerebellar activity via transcranial magnetic stimulation (TMS) abolished the adaptation of M100 attenuation, while the M200 attenuation reverses to an M200 enhancement. Our results provide causal evidence for the involvement of the cerebellum in adapting to delayed action effects, and thus in the prediction of the sensory consequences of our actions.

## Highlights

• Adaptation of M100 attenuation for delayed action effects was localized in cerebellum
• Cerebellar stimulation abolished the adaptation of M100 attenuation
• M200 attenuation reversed after cerebellar stimulation
• Cerebellar low-frequency activity may be important for forward model updating

## Teaser

Cao et al. show that the adaptation to delayed action effects disappears after an inhibitory cerebellar stimulation with TMS, highlighting the cerebellum’s role in forward model updating. The stimulation also changes cerebellar low-frequency activity and evokes responses in the frontal brain area, suggesting a processing hierarchy for prediction errors.

## Introduction

Self-generated stimuli are ubiquitous in everyday life. As I am typing these words, every stroke on the keyboard generates predictable visual (i.e., the character), somatosensory (i.e., the fingertip tap), and auditory (i.e., the keyboard click) consequences. Such sensory events resulting from voluntary actions elicit smaller brain responses as compared to the same events when they are externally generated. This phenomenon is known as sensory attenuation [1, 2]. For example, the amplitude of early cortical responses to a tone peaking at around 100/200 ms after sound onset (known as M100/M200 components in magnetoencephalography [MEG] recordings) is smaller for self-generated tones than for external tones [1, 2, 3, 4]. When a perturbation is introduced between actions and the ensuing sound (e.g., by adding a delay between an action and the onset of the tone), sensory attenuation is reduced or even abolished in the case of a large perturbation [5, 6]. Yet, the brain can adapt to small perturbations so that the sensory attenuation effect re-emerges after learning [5, 7]. Aliu and coworkers [5] showed that when a tone was delivered with a 100-ms delay after a button press, the auditory sensory attenuation in the M100 component was initially absent but re-emerged during learning within 300 trials. Learning-related changes of the M200 attenuation have not been studied to the best of our knowledge.

Sensory attenuation can be explained as the consequence of computations by an internal forward model [3, 8]. The forward model theory posits that predictions for the sensory consequences (including its timing) of an action are formed along with the motor command that will elicit this action. When the reafferent signal from self-generated stimuli reaches the brain, it is compared to this prediction and sensory attenuation will be observed if the real sensory input and the prediction match. Any mismatch (prediction errors) will be relayed to a higher-order brain area for further processing [9]. With this in mind, the process of sensory attenuation re-emerging after perturbation can be seen as a process of correcting previous predictions to account for the perturbation or a process of updating outdated forward models to minimize prediction errors. Interestingly, recent studies suggest that the cerebellum may play a vital role in the updating of forward models. For example, cerebellar lesion patients were found to show deficits in predicting the position of self-controlled cursor on the screen when a discrepancy was introduced between the real cursor position and the controlling movement [10, 11]. There is also evidence suggesting the involvement of the cerebellum in temporal adaptation [12, 13, 14]. However, temporal information about stimulus appearance was provided by the stimulus context in these previous studies (e.g., through rhythmic stimulus presentation). Whether the cerebellum will also be involved in the adaptation when the temporal information is provided by self-action is an open question. Given the role of the cerebellum in the forward model updating and in representing temporal prediction errors for self-generated stimuli (e.g., [15]), this is likely. Here, linking action and perception, we seek causal evidence for the involvement of the cerebellum in learning to predict sensory consequences of our actions (when there is a delay between action and sensory input) in the healthy population.

**Figure 1:**
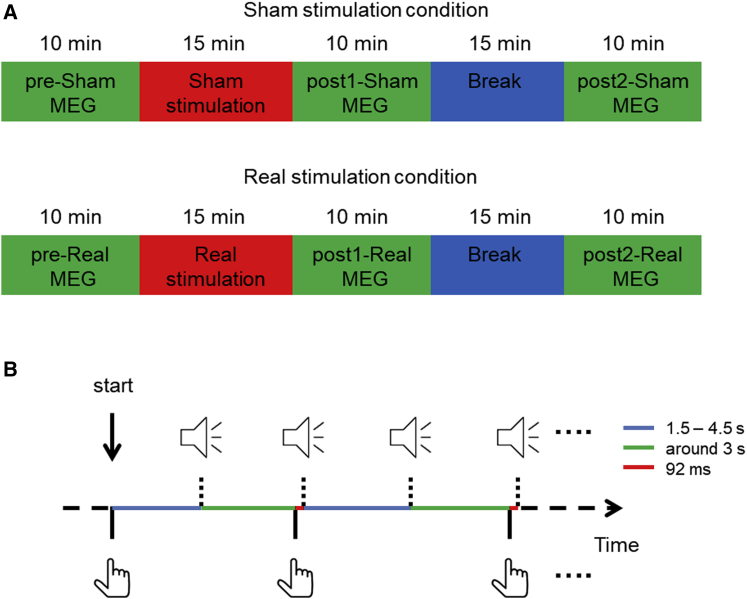
Testing Procedure (A) Schematic representation of the testing procedure for both real and sham stimulation conditions (at least 6 days apart). (B) Schematic representation of the behavioral task used during MEG recordings. A finger lift marks the beginning of testing, which is followed by an external tone. After hearing the external tone, participants are instructed to wait about 3 s to make a finger lift to trigger a self-generated tone. The self-generated tone is delayed for presentation by 92 ms on average. Then a new external tone is presented after a random interval between 1.5 and 4.5 s. Self-generated tones and external tones are played alternatingly in this way until 200 total tones are played.

To investigate the process of forward model updating, we used an auditory sensory attenuation paradigm and introduced a delay between actions and action-associated tones, while non-invasively recording brain activity using MEG. We employed the identical auditory sensory attenuation paradigm in three consecutive MEG testing sessions over 2 days (Figure 1). The first session served to measure the baseline sensory attenuation effect. Next, we transiently suppressed normal cerebellar function using transcranial magnetic stimulation (TMS) to probe for cerebellar involvement in updating of the forward model. For this purpose, we utilized an inhibitory offline 1 Hz-repetitive (r)TMS protocol that is known to induce a lasting suppression of brain activity [16], and we compared these effects to sham TMS sessions recorded on a different day. TMS stimulation (sham or real) was immediately followed by a second MEG session. After a 15-min break, a third MEG recording was performed. Each MEG session took about 10 min. The order of active TMS session and sham session was counterbalanced.

We expected to replicate (1) the M100 and M200 sensory attenuation effect at baseline (first session) as well as (2) the adaptation of M100 attenuation over time to the introduced delay between action and its sensory consequence (i.e., the tone), which should manifest over sham sessions as unperturbed learning occurs. We also predicted that (3) real cerebellar stimulation would interfere with this M100 attenuation adaptation. In addition, we aimed to (4) explore the effect of learning on M200 attenuation effect under both normal and perturbed cerebellar functioning and (5) source localize the M100 and M200 changes. Finally, we hypothesized that (6) cerebellar contributions are expressed in or mediated by low-frequency activity before stimulus presentation in the cerebellum, since low-frequency oscillations are known to reflect cyclic excitability changes in neuronal populations [17].

## Results

### Replication of M100 and M200 Sensory Attenuation Effects in Baseline Sessions

**Figure 2:**
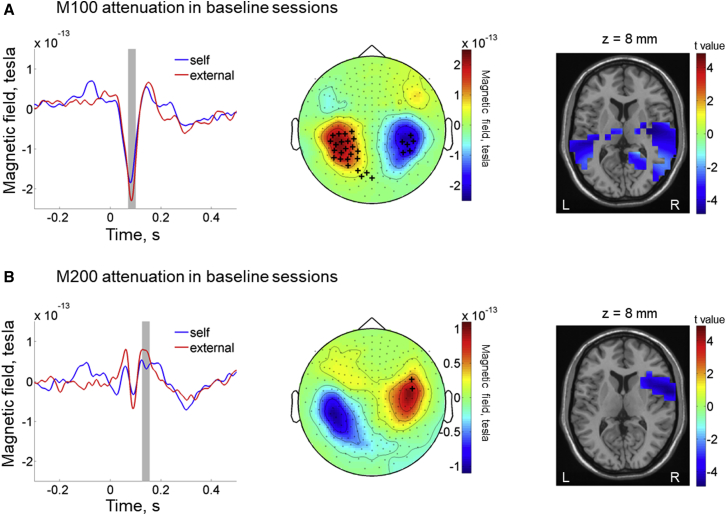
Replication of Sensory Attenuation in Baseline Sessions (A) Average evoked responses (left panel) are plotted from all the right hemisphere sensors (middle panel) where significant M100 attenuation is found (sensors marked with a plus sign; p < 0.05 with paired t tests; no correction for multiple comparisons). Background color in the middle panel shows the M100 topography (70–100 ms; grand average of the external tone-evoked responses in the pre-real session). The results are illustrated with the data from the pre-sham session. Source localization results with combined data from both baseline sessions (pre-sham and pre-real) show that M100 attenuation in the baseline session is strongest in the bilateral auditory cortex (right panel). *Z*-coordinate score is referenced to the Montreal Neurological Institute (MNI) brain. L and R indicate left and right, respectively. (B) The results of M200 attenuation in pre-real session are shown (left and middle panels) similarly to M100 attenuation effect. Background color in the middle panel shows the M200 topography (125–155 ms; grand average of the external tone-evoked responses in the pre-real session). Source localization results from combined baseline session data (pre-sham and pre-real) show that the strongest M200 attenuation is in the inferior frontal cortex and insula (right panel).

In electroencephalography (EEG) studies, sensory attenuation is typically indexed by an amplitude reduction of N100 and P200 components for self-generated tones in comparison to externally generated tones [1, 2, 3]. Here we focused on their MEG counterparts: the M100 and M200 components. Our analysis demonstrated significant M100 and M200 attenuation for self-generated tones as compared to external tones (paired t tests, all p < 0.05) in the first testing session (i.e., at baseline) at or close to sensors showing the strongest auditory evoked responses (see sensors marked with plus sign in Figure 2). Source localization on the pooled data from both baseline sessions (pre-sham and pre-real) showed that M100 attenuation was strongest in bilateral temporal auditory areas (Figure 2A) and M200 attenuation was strongest in inferior frontal gyrus and insula (Figure 2B).

### Replication of M100 Adaptation: Sensory Attenuation Amplifies over Testing Sessions in Sham Stimulation Condition

**Figure 3:**
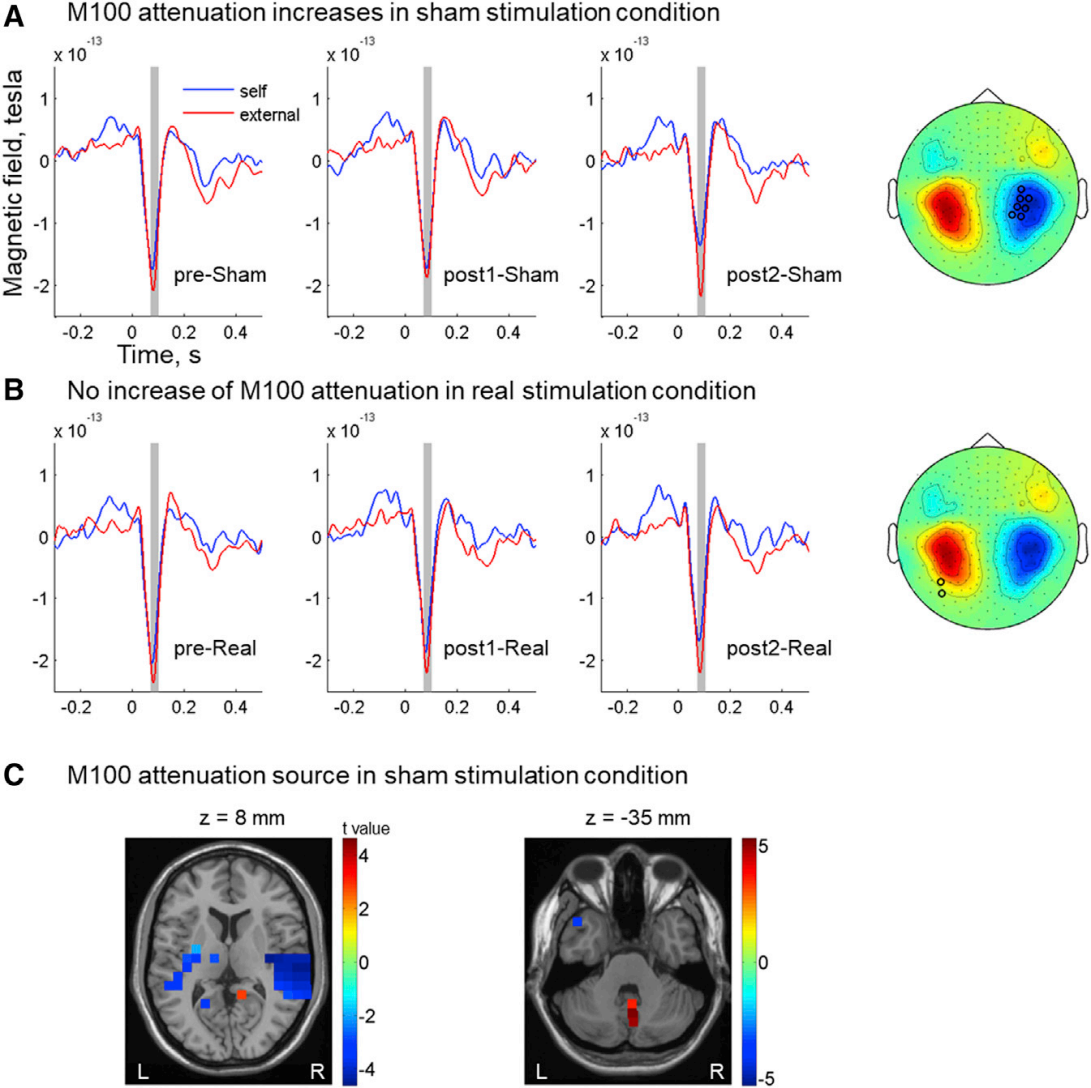
M100 Attenuation over Testing Sessions (A and B) A significant increase in M100 attenuation over testing sessions is found in the sham stimulation condition (A), but not in the real stimulation condition (B). Sensors showing significant results (marked with open circles; p < 0.05 without correction for multiple comparisons) from the within-subject ANOVA of M100 attenuation over testing sessions are marked in the M100 topography plot. The evoked responses are plotted from the two sensors showing the largest F values in (A). The shaded area shows the time window of the M100 component (70–100 ms post-stimulus) selected for analysis. (C) Voxels showing significant M100 attenuation (strongest in bilateral auditory cortex) in the pre-sham session are marked with blue color, and voxels showing a significant increase in M100 attenuation (in cerebellar vermis) over sessions are marked with red color. Voxels showing both attenuation and changes of attenuation are marked with cyan color (equivalent to a value of 0). Color bars represent t values except for the value 0. In this case, no overlap between the two effects (i.e., no cyan color) is found. *Z*-coordinate score is referenced to the MNI brain. See also Figures S1 and S2.

To analyze M100 adaptation, we performed a within-subject one-way ANOVA analysis on the M100 attenuation from the three testing sessions for the sham condition only. As hypothesized, this revealed a significant increase in M100 attenuation (p < 0.05; Figure 3A) over testing sessions. This indicates an adaptation to the delay between the action and its sensory consequence that was implemented here. The increased sensory attenuation effect was specific to the M100 component and specific to the sham stimulation condition (see Figure S1A). Interestingly, source localization analysis showed the strongest increase in M100 attenuation in the sham condition in the cerebellar vermis (Figure 3C). Bilateral precuneus showed also a significant but less pronounced increase in M100 attenuation. No auditory areas in the temporal cortex showed a significant increase in M100 attenuation.

To further investigate the origin of the adaptation effect (increased M100 attenuation) during sham sessions (i.e., an amplitude decrease for self-generated tones versus an amplitude increase for external tones), a two (self-generated versus external) by three (testing sessions: pre-sham, post1-sham, and post2-sham) within-subject ANOVA was performed on the amplitude of M100 components. This revealed a significant interaction effect (F(2,18) = 5.35, p = 0.02). Post hoc analysis showed a reduction in M100 amplitude for self-generated tones in post2-sham session compared to external tones in post2-sham session (t(9) = 2.57, p = 0.03) and self-generated tones in post1-sham session (t(9) = 2.44, p = 0.04). The first simple effect hence confirms the presence of a significant M100 attenuation effect in post2-sham. The latter simple effect reveals that its adaptation over time is due to a decrease in M100 amplitudes for self-generated tones. No other effects from the ANOVA analysis reached statistical significance (main effect of tones: F(1,9) = 3.77, p = 0.08; main effect of testing session: F(2,18) = 0.26, p = 0.78). See Figure 3 for an illustration of M100-evoked responses in all testing sessions. This analysis therefore confirmed the predicted increase in M100 attenuation due to adaptation to the delay between an action and its sensory consequence. As expected, the change in M100 attenuation is due to the reduced M100 response for self-initiated sensory stimuli.

### Interference of Cerebellar TMS with M100 Adaptation

**Figure 4:**
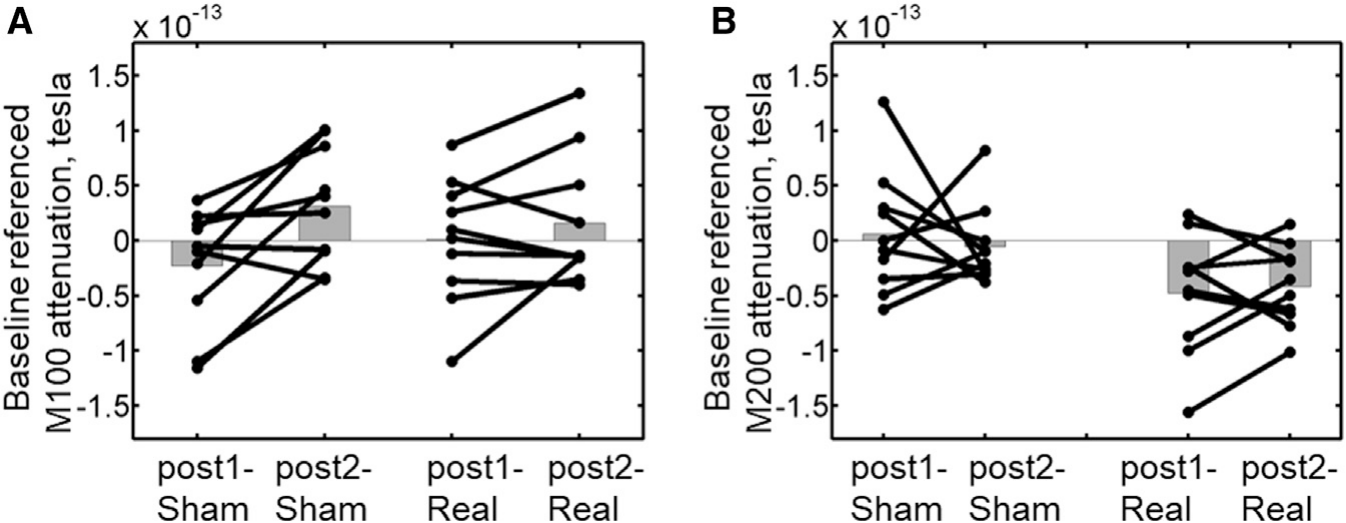
Results of Baseline-Referenced M100 and M200 Attenuation Effects (A) The two (sham versus real stimulation) by two (post1 versus post2) ANOVA on the baseline-referenced M100 attenuation effect reveals a significant interaction effect (p = 0.02). There is a significant increase in M100 attenuation from post1-sham to post2-sham (p = 0.01), but not from post1-real to post2-real (p = 0.27). Individual results are overlaid on the group means shown by the gray column. (B) The two (sham versus real stimulation) by two (post1 versus post2) ANOVA on the baseline-referenced M200 attenuation effect reveals a significant main effect of stimulation condition (p = 0.03). The M200 attenuation effect is significantly smaller in real stimulation condition than in sham stimulation condition. On the contrary, reaction time did not show similar modulation by the stimulation (see Table S1).

Having shown the adaptation of M100 attenuation in the sham stimulation condition, we next investigated whether cerebellar TMS affected M100 attenuation across sessions, as compared to the sham condition. Since no significant difference (paired t test, t(9) = 0.13, p = 0.90) was found between pre-sham and pre-real M100 attenuation (pre-sham mean = 3.53e−14, SD = 5.42e−14; pre-real mean = 3.34e−14, SD = 5.81e−14) (Figures 3A and 3B, left panel), each post-stimulation M100 attenuation effect was referenced to the corresponding baseline (pre-stimulation) M100 attenuation effect (through subtraction) before being subjected to a two (sham versus real stimulation) by two (post1 versus post2) within-subject ANOVA analysis (see Figure 4A for baseline-corrected data). This analysis showed a main effect of testing session with an increase inbaseline-referenced M100 attenuation (F(1,9) = 7.21, p = 0.03) from post1 (mean = −1.11e−14, SD = 2.81e−14) to post2 (mean = 2.36e−14, SD = 3.80e−14). Importantly, there was a significant interaction between stimulation condition and testing session (F(1,9) = 7.71, p = 0.02). Post hoc analysis revealed a significant increase in M100 attenuation (t(9) = 3.31, p = 0.01) from post1-sham (mean = −2.31e−14, SD = 5.35e−14) to post2-sham (mean = 3.11e−14, SD = 5.25e−14) but no significant change (t(9) = −1.19, p = 0.27) from post1-real (mean = 8.64e−16, SD = 5.69e−14) to post2-real (mean = 1.61e−14, SD = 5.85e−14). These results demonstrate that the inhibitory cerebellar TMS protocol abolished the adaptation of M100 attenuation.

### M200 Sensory Attenuation: Adaptation and TMS Effects

**Figure 5:**
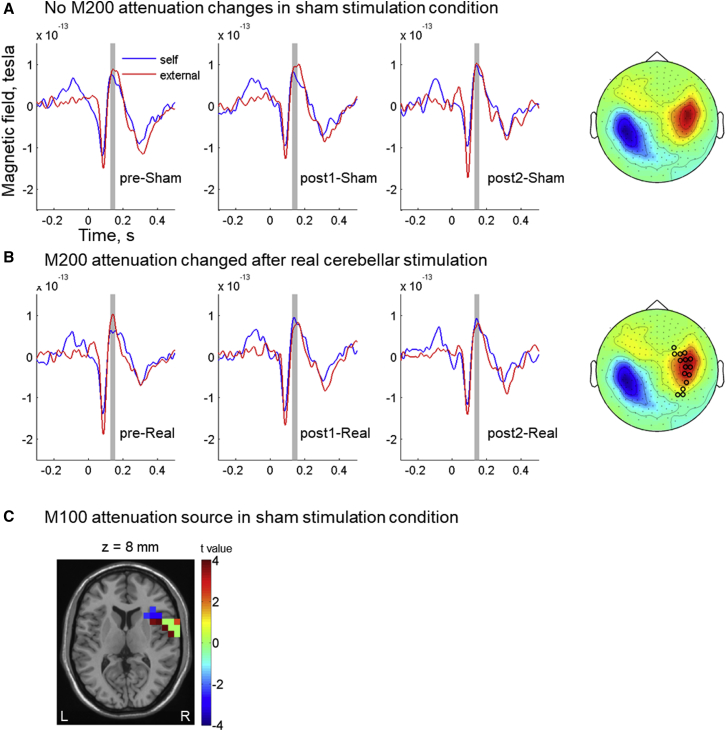
M200 Attenuation over Testing Sessions (A and B) Significant changes of M200 attenuation over testing sessions are not found in the sham stimulation condition (A), but they are found in the real stimulation condition (B). Sensors showing significant results (marked with open circles; p < 0.05 without correction for multiple comparisons) from the within-subject ANOVA of M200 attenuation over testing sessions are marked in the M200 topography plot. The evoked responses are plotted from the two sensors showing the largest F values in (B). The shaded area shows the time window of the M200 component (125–155 ms post-stimulus) selected for analysis. (C) Voxels showing significant M200 attenuation in the pre-real session are marked with blue color, and voxels showing significant changes of M200 over sessions are marked with red color, both of which have strongest signals in the right inferior frontal gyrus and insula (indicated by cyan color). Color bars represent t values except for the value 0. *Z*-coordinate score is referenced to the MNI brain. See also Figures S1 and S2.

To explore adaptation and TMS interference for the M200 component, the data were subjected to the same analyses as for the M100 component. The within-subject one-way ANOVA analysis on the sham data revealed that there was no adaptation of the M200 attenuation effect over time (Figure 5A; p > 0.05). However, a modulatory effect on M200 attenuation was observed for the real TMS condition (Figure 5B; see also below and Figure S1B). In contrast to the M100 component, source localization revealed that the brain area showing the strongest changes in M200 attenuation from TMS (Figure 5C) overlapped with the brain area where M200 attenuation effect was significant in the baseline session (i.e., inferior frontal gyrus and insula).

To further examine the effect of cerebellar TMS and in keeping with our analysis approach for the M100 component, we subjected the M200 attenuation effect to a two (sham versus real stimulation) by two (post1 versus post2) within-subject ANOVA analysis after being referenced to the M200 attenuation effect found during the baseline session (through subtraction), for which no significant differences were found (t(9) = −1.58, p = 0.15) between the sham stimulation condition (Figure 5A, left panel; mean = 1.17e−14, SD = 5.93e−14) and the real stimulation condition (Figure 5B, left panel; mean = 3.27e−14, SD = 6.23e−14). The two-by-two ANOVA revealed a significant main effect of stimulation condition (F(1,9) = 6.69, p = 0.03), with the baseline-referenced M200 attenuation effects being significantly smaller in real stimulation condition (mean = −4.45e−14, SD = 4.14e−14) relative to the sham stimulation condition (mean = 3.53e−16, SD = 3.05e−14) (Figure 4B). Specifically, after real stimulation, the M200 amplitude was numerically higher for self-generated than for external tones, indicating that the baseline sensory attenuation was abolished if not inverted (M200 enhancement).

A final two (self-generated versus external) by three (testing sessions) within-subject ANOVA analysis of the amplitude of M200 components in real stimulation condition was performed, and it revealed a significant interaction effect (F(2,18) = 6.74, p = 0.01). However, no simple effects reached statistical significance in post hoc analysis. See Figure 5 for an illustration of M200-evoked responses in all testing sessions.

### TMS Modulates Low-Frequency Pre-stimulus Activity in the Cerebellum

**Figure 6:**
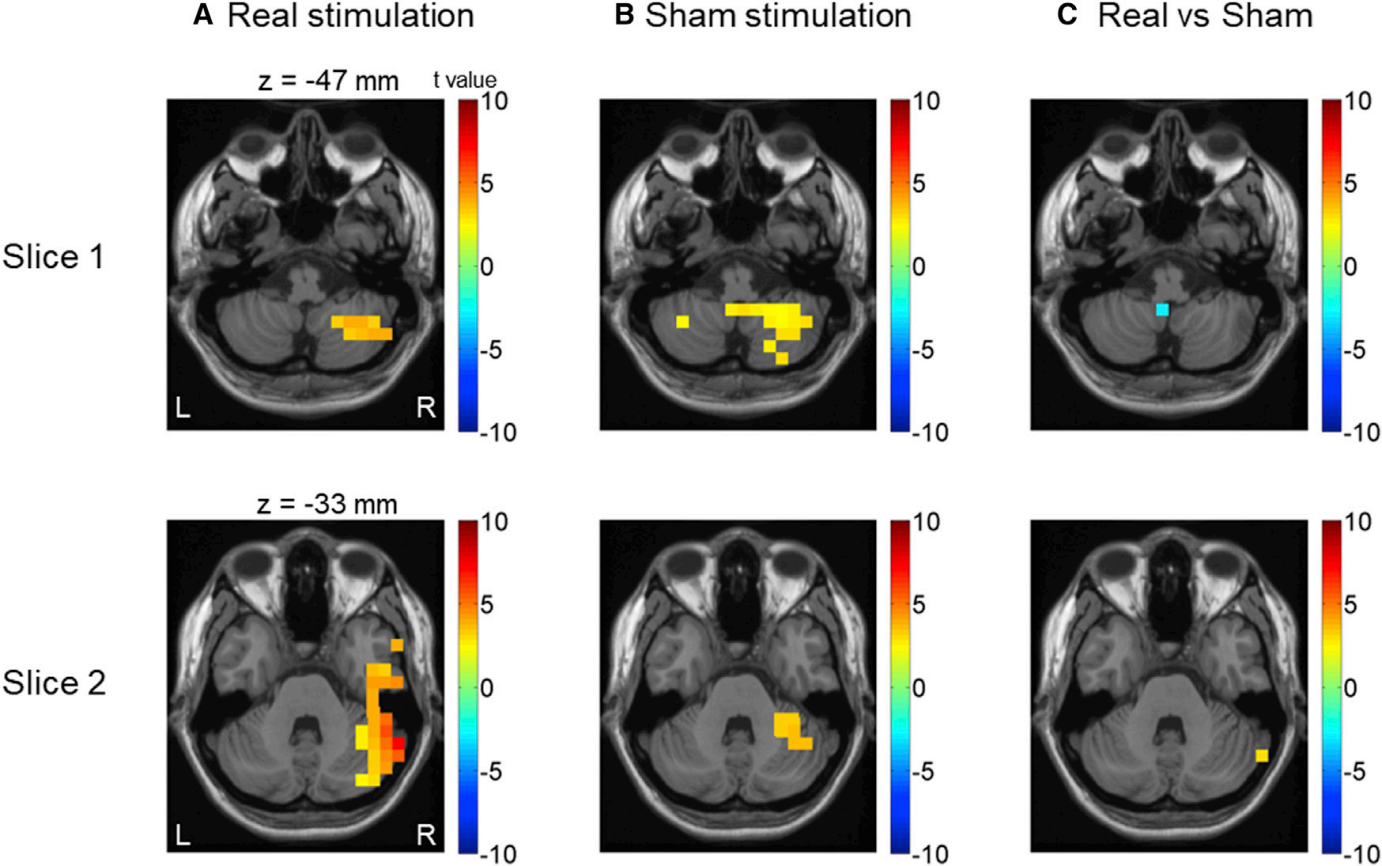
Significant Changes of Pre-stimulus Low-Frequency Activities in the Cerebellum In this exploratory analysis, pre-stimulus (−500–0 ms) cerebellar low-frequency (below 10 Hz) activities were compared for trials with self-generated tones between post and pre sessions in the real (A) and sham stimulation conditions (B). In real stimulation condition, a significant increase in pre-stimulus low-frequency activity is only found in the right cerebellar hemisphere (close to the stimulation site). But in sham stimulation condition, pre-stimulus low-frequency activity increases in both the right cerebellar hemisphere and cerebellar vermis. Significant differences between real and sham stimulation conditions are found in both the right cerebellar crus and cerebellar vermis (C). *Z*-coordinate score is referenced to the MNI brain.

Finally, we explored the idea that the cerebellar TMS effect on adaptation to delayed action effects may be associated with TMS modulating low-frequency oscillatory activity. To test this hypothesis, we used pre-stimulus data that were not contaminated by auditory-induced activity. We first compared the single-trial source-reconstructed pre-stimulus time series (−500–0 ms; 10-Hz low pass) between the two post-TMS sessions and the pre-TMS session to assess the development of low-frequency activity over testing sessions, separately for real and sham stimulation conditions. Then the real and sham stimulation conditions were compared to identify changes induced by TMS. An interesting difference emerged from this comparison in trials for self-generated sounds between the real and the sham stimulation conditions. The right hemisphere cerebellar crus (close to the stimulation site) showed significantly stronger activity, and the cerebellar vermis (close to the site of M100 attenuation increase in the sham stimulation condition) showed significantly reduced low-frequency activity in real stimulation as compared to sham stimulation condition (Figure 6). No such difference was found in trials for externally generated sounds. A close examination indicated that pre-stimulus low-frequency activities increased more in the right hemisphere cerebellar crus in post-real sessions than in post-sham sessions. In the cerebellar vermis, pre-stimulus low-frequency activities remained unchanged in the real stimulation condition, but they increased in the sham stimulation condition. These findings suggest that the inhibitory TMS protocol induces changes in low-frequency activity in the cerebellum.

### Control Analysis

We performed additional analysis to rule out that the motor response, which is associated with self-generated tones, but not with external tones, may contribute to our sensory attenuation results (see also the Discussion). First, a time domain cluster analysis in the baseline time window (−600 to −200 ms) comparing self-generated tones and external tones in the pre-TMS session did not yield any significant results on the sensors showing the M100 and M200 attenuation effects (see Figure S2). This demonstrates that sensory attenuation in our data was not caused by differences in the time window used for baseline correction (−600 to −200 ms). Second, we also analyzed the timing of self-generated tones between button presses across conditions (sham versus real) to rule out that differences in timing of these manual responses (which would differentially affect session durations) may be associated with the observed sensory attenuation changes. No significant interaction between stimulation condition (sham versus real) and testing session (pre, post1, and post2) was found (Table S1). This provides evidence that changes in auditory-evoked responses over testing sessions are not caused by changes in response latency.

## Discussion

In this combined TMS-MEG study, we provide first-time evidence of a causal role of the cerebellum in adapting to delayed action effects in the auditory domain in healthy young participants. Regarding the M100 component, we replicated previous findings of sensory attenuation for self- versus externally generated tones at baseline and of an increase in M100 attenuation within 300 trials of learning to adapt to a 100-ms delay between action and tone [5, 7]. Source localization showed that the increase in M100 attenuation was strongest in the cerebellum. After we stimulated the cerebellum with an inhibitory TMS protocol, the increase in M100 attenuation disappeared. For the M200 component, in contrast, we did not observe an adaptation of sensory attenuation over trials, but we demonstrated an inversion of M200 attenuation to M200 enhancement by cerebellar TMS. This change of M200 attenuation was strongest in inferior frontal gyrus and insula. Further analysis provided preliminary evidence that low-frequency activity (below 10 Hz) in the cerebellum prior to stimulus presentation plays a key role in adapting to delayed action effects. Overall, this extends the role of the cerebellum in updating forward models from the visual to the auditory domain [10, 11].

The forward model associated with actions has been shown to modulate sensory cortices through top-down mechanisms [4]. When a self-generated stimulus activates sensory areas, the brain’s responses result from a complex interplay between the bottom-up and top-down signals. Our findings show that the M100 and M200 components represent functionally and anatomically distinct neural information-processing stages in this process (see below for details). Moreover, our design combining two non-invasive tools in cognitive neuroscience (TMS and MEG) allows gaining valuable insights into the role of the cerebellum in updating the forward model when the predicted signal does not match the reafferent signal (discussed below).

A decrease in the amplitudes of M100 and M200 components (and their EEG counterparts N100 and P200 components) for self-generated tones is a very robust effect, reliably demonstrating the influences that actions can have on auditory processing [1, 2, 4, 18, 19]. In addition, many studies demonstrated a functional disassociation between the two components [19, 20, 21, 22, 23], which is also supported by our results showing different characteristics of both components over testing sessions in sham and real stimulation conditions. The M100 attenuation in the baseline session (pre-sham and pre-real) was localized in auditory cortex, which we interpret as a result of predictions from the forward model. Our results also show that the forward model is tolerant, to some degree, to a delay in the stimulus onset [18]. Still, within 300 trials of learning, M100 attenuation is further increased, reflecting updated predictions that take into account the temporal delay between action and tone. Two main results from our analysis demonstrate a significant and causal involvement of the cerebellum in this process. First, the increase in M100 attenuation over sessions (reflecting the learning of the action-tone delay) was only evident in the sham condition but abolished after cerebellar TMS. The particular TMS protocol used here (1 Hz-rTMS, 900 pulses) has been reported to interfere with brain activity for up to 40 min following stimulation offset [16]. Therefore, we can assume that learning-related processes that rely on cerebellar functions were significantly impaired for the two MEG sessions following real TMS. Our results are therefore consistent with a critical role of the cerebellum in the implementation or mediation of the learning-related changes of sensory attenuation. Second, our source localization results reveal the strongest changes of M100 attenuation over sessions in the cerebellum. We acknowledge that MEG has a greatly reduced sensitivity to activity originating from subcortical compared to cortical brain areas. Still, under favorable conditions, subcortical brain areas in general and cerebellar areas in particular have been successfully studied with MEG [24, 25, 26]. Our results are also in line with findings suggesting that the cerebellum is important for sub-second timing accuracy [27, 28]. Consequently, when normal cerebellar function was suppressed by TMS, the increase in M100 attenuation over sessions reflecting adaptation of the forward model disappeared (Figure 3).

M200 attenuation showed a different pattern. We suggest that the M200 component represents processing at a higher level in the auditory-processing hierarchy compared to the M100 component and that the M200 component aims to deal with residual prediction errors not resolved in earlier processing stages. Thus, in sham stimulation condition, M200 attenuation may have remained unchanged as the prediction error resulting from the delay was already resolved by the cerebellum. When TMS interfered with cerebellar function in the real stimulation condition, the prediction error was not resolved by the cerebellum; it still existed at later processing stages in inferior frontal gyrus and insula, and it was reflected in a relative increase in the amplitude of M200 component. This explanation of M200 component is supported by other studies showing that the M200 amplitude (or P200 in EEG studies) increases when a stimulus cannot be predicted [29] and when a predicted stimulus is omitted [30] or violated [31]. This may also explain why, in EEG studies, N100 attenuation, but not P200 attenuation, was observed in the following two cases: (1) when a stimulus was followed by actions of an atypical effector, like the eye [19] or the foot [21], and (2) when a non-speech stimulus followed speech movement planning [22]. In summary, M100/N100 attenuation may be the result of predictions from forward models that act on low-level features of the stimulus. Prediction errors from a higher-level comparison will be further addressed in inferior frontal gyrus and insula in the time window of the M200/P200 component, which may be used for guiding future predictions [32].

Numerous theoretical and empirical studies have implicated the cerebellum in the neural circuit of the forward model [33]. These studies suggest that the cerebellum is involved in all steps of the forward model from making predictions [20, 34] and encoding prediction errors [15, 35, 36] to updating forward models [10, 11]. But how the cerebellum performs these functions is largely unknown. Here we show that the cerebellum is involved in adapting to the delay between actions and action-induced sounds, i.e., updating the forward model. It is well known that the brain has a temporal integration window during which a motor act and its associated stimulus (or between two separate stimuli in multisensory research) are perceived simultaneously [37, 38]. It can be envisaged that, following a motor act (or the first stimulus of a pair of stimuli), the brain has a probabilistic distribution model of the predicted arrival time of the associated stimulus (probably with a window width of around 200 ms depending on the context). If the arrival time of the stimulus falls within the range of the predicted distribution window, the sense of simultaneity and agency can be formed and an update of the distribution model is triggered depending on the mismatch between the stimulus arrival time and prediction. In this case, the update of the distribution model involves the cerebellum, which is known to be engaged in sub-second temporal information processing [27, 39, 40], and no conscious awareness of the delay is formed, i.e., without involvement of higher cognitive brain areas. If the stimulus arrives out of the range of the predicted window, the stimulus will not be perceived as simultaneous to the motor act and the sense of agency toward the stimulus will be compromised. Note that the action-stimulus association can still be formed, but whether the cerebellum is involved is unclear [18, 41].

Prediction and error-based learning are key concepts in cerebellum research [42]. In the current study, the predicted arrival time of the stimulus is provided by a motor act. It is unknown if the abovementioned probabilistic distribution model of stimulus arrival time is implemented in the cerebellum, cerebral cortex, or both. In any case, the prediction error resulting from the stimulus arrival time is available to the cerebellum locally or possibly through cortico-cerebellar connections [43, 44], thus allowing for the forward model updating. In a time window preceding stimulus onset, significant changes in low-frequency signals between the real stimulation condition and the sham stimulation condition were only observed for trials of self-generated sounds, and the M100 amplitude decrease was only observed for self-generated sounds in the real stimulation condition. This suggests an important role of low-frequency oscillations in updating the forward model. The prediction is still available to the cerebellum after disruption by TMS, since the M100 attenuation effect after stimulation is not significantly different from the baseline session, which is consistent with other lines of research [12]. As a consequence, the failure to update the forward model could be due to either the prediction error not being available or the updating mechanism being impaired or both. Our results cannot distinguish among these possibilities. Interestingly, theoretical models posit that the cerebellum is sensitive to stimulus arrival time and low-frequency neural oscillations may be involved in the calculation of temporal intervals [17, 40, 45]. Accordingly, it is conceivable that, in the current study, the prediction error (the interval between the predicted stimulus arrival time and the real stimulus arrival time) was not well processed after real cerebellar stimulation, as the pre-stimulus low-frequency signals increased less in the real stimulation condition than the sham stimulation condition in the cerebellar vermis. How low-frequency oscillations coordinate cerebellar functioning is an intriguing question for further study.

Another interesting question is the duration required for adapting to delayed sensory consequences of actions. Our results indicate that the adaptation to 100-ms-delayed tones takes more than 100 trials, as revealed in the neuromagnetic signals (see also [5, 7]), whereas previous behavioral studies demonstrated a related temporal recalibration effect with a very few number of trials (within 20 trials or even less depending on the paradigm) [37, 38, 46]. For example, Stetson et al. [37] demonstrated that participants can quickly adapt to a short-delayed (100-ms) action effect so that, after the adaptation, they tend to report an even shorter delayed action effect (e.g., 40 ms) as happening before the button press. There are several possibilities for this discrepancy in the time course of adaptation. First, there are critical differences between our study and those previous behavioral studies. Our study addressed the question of neural temporal adaptation in the simplest form (i.e., as a passive task), whereas a timing task (e.g., judging whether the action effect is before or after the action) is always required in behavioral studies for measuring the adaptation effect. The involvement of behavioral tasks might have facilitated the adaptation process. Second, our study revealed changes in low-frequency activity in a pre-stimulus interval, which we interpret to reflect a prediction component in the updating of temporal prediction, which may or may not be present in behavioral studies. These studies rely on behavioral responses to infer the underlying effect, which make them open to explanations not related to prediction-related processes. For example, participants may perform the temporal judgement task by using the somatosensory feedback as a reference point, and a shift of perceived onset time of action may explain the temporal recalibration effect [47]. Interesting questions for future studies are whether there is a behavioral correlate of our results and how individual differences in sensory attenuation (and its changes) relate to individual task performance. A possible idea is to adapt the current task to a behavioral sensory attenuation task (therefore, prediction will be involved) and investigate the temporal evolution of the task performance.

Finally, differences in design compared to other sensory attenuation studies need to be discussed. Several previous studies on sensory attenuation in EEG/MEG activity have implemented a motor control condition in which a motor act is performed without stimulus presentation (e.g., [2, 18, 19]). The evoked response in the motor control condition is then subtracted from the auditory-evoked responses with self-generated sounds to correct for any potential motor contamination in the auditory-evoked responses due to the motor act preceding the stimulus presentation (however, see [48] for negative evidence of the effectiveness of this strategy). We did not include such a control condition to avoid making the study excessively long, because of the focus of our study on adaptation (see also [49, 50] for examples of other MEG sensory attenuation studies not using such a control condition). Most importantly, a motor contamination explanation of our findings can be ruled out from the timing and spatial distribution of our results. First, our results are exclusively based on sensors picking up auditory responses (Figures 3 and 5) in their typical time windows (Figure S1). Second, source localization demonstrates main effects in auditory areas. This particular spatiotemporal pattern provides evidence against an explanation based on motor contamination because of not overlapping in space or time with the motor-related activity of the task. Third, a control analysis showed that the baseline time window prior to sound presentation (which may have been associated with motor contaminations) did not contribute to sensory attenuation, further ruling out motor contamination as an explanation of our results. In further contrast to some other sensory attenuation studies, we also did not include a condition in which the sound is presented with a 0-ms delay, which would have provided a useful condition for estimating individual sensory attenuation in the absence of a delay. However, we compared magnitudes of M100 attenuation between the current study and our previous MEG study [4] in which a similar paradigm was used but without delay between the motor act and sound presentation. In sham stimulation condition of the current study, the M100 attenuation effect in the baseline session was around 50%, and in the last testing session it was around 100% of the M100 attenuation effect measured without a delay. Importantly, we showed a reduction in amplitudes of evoked responses across sham sessions for self-generated tones, but not external tones, which indicates that the delay between the motor action and sound presentation was taken into account over time. Together with other studies [5, 7], our results therefore provide converging evidence that the adaptation to a delay between a motor act and its associated sensory consequence can be revealed in electromagnetic-evoked responses at around 100 ms post-stimulus.

In summary, our study provides conclusive evidence for a causal involvement of the cerebellum in the updating of internal forward models related to the process of predicting (temporal) sensory consequences of actions.

## STAR★Methods

### Key Resources Table

**Table:** 

REAGENT or RESOURCE	SOURCE	IDENTIFIER
**Software and Algorithms**		

MATLAB	The MathWorks	https://www.mathworks.com/products/matlab.html
FieldTrip toolbox	[51]	http://www.fieldtriptoolbox.org/

### Contact for Reagent and Resource Sharing

Further information and requests for resources and reagents should be directed to and will be fulfilled by the Lead Contact, Liyu Cao (liyu.cao@uni-wuerzburg.de).

### Experimental Model and Subject Details

Ten healthy, right-handed volunteers (including LC; 5 males; mean age = 23.0, SD = 2.7) were recruited from a local participants’ pool. Participants gave written informed consent prior to the experiment and received monetary compensation after the experiment. None of the participants had any contraindication to TMS or any neurological, psychiatric, or other relevant medical condition [52]. The study was approved by the local ethics committee (Ethics Committee of College of Science and Engineering, University of Glasgow) and was conducted in accordance with the Declaration of Helsinki.

### Method Details

#### Equipment

A double-cone TMS coil connected to a MagStim Rapid^2^ magnetic stimulator (The Magstim Company, Whitland, UK) was used for stimulating the right cerebellum. This type of coil was used because it has been demonstrated to be the most effective for cerebellar stimulation when compared to other coils [53]. A 248-magnetometers whole-head MEG system (MAGNES 3600 WH, 4-D Neuroimaging) was used for MEG data recording with a sampling rate of 1,017Hz.

#### General Procedure

Each participant was tested in a real stimulation and a sham stimulation condition on different days (at least 6 days apart). In each stimulation condition (Figure 1A), sensory attenuation effect was measured three times using the same procedure (see ‘MEG measurements’ section below) and the stimulation was performed after the first sensory attenuation measurement. After baseline sensory attenuation measurement (pre-Sham/pre-Real), participants were encouraged to take a break, after which the rTMS stimulation was performed outside the magnetically shielded room. The second sensory attenuation measurement (post1-Sham/post1-Real) started just after the stimulation. The delay between the end of rTMS and the start of second sensory attenuation measurement was comparable (t(9) = −0.97, p = 0.36) between real (mean = 2.60; SD = 0.38; in minutes) and sham stimulation condition (mean = 2.92; SD = 1.09; in minutes). The delay between the end of the second sensory attenuation measurement and the start of the third sensory attenuation measurement (post2-Sham/post2-Real) was always about 15 min (about 25 min after TMS stimulation) for each condition and each participant.

#### Transcranial Magnetic Stimulation

A 15 min inhibitory 1 Hz repetitive stimulation protocol (1Hz-rTMS) was used. Initially, the cerebellar stimulation location was set using the same scalp co-ordinates as in [54], 1cm below and 3cm to the right of the Inion. The scalp coordinates were then projected onto individual’s structural MRI (obtained at least 6 days before any MEG recordings) via Brainsight (Rogue Research) to ensure effective targeting of the cerebellum. In 5 participants, the stimulation location was then moved down by ∼0.5 cm from the initial point as its projection was localized in between the occipital cortex and the cerebellum. The final stimulation point was determined prior to the first sensory attenuation measurement. The stimulation intensity was set at 50% of maximum stimulator output; however, in 7 of the 10 tested participants the intensity was adjusted to reduce discomfort caused by muscular activation (mean stimulation intensity in 10 participants = 46.5%; post hoc check showed that the stimulation intensity was not correlated with main effects reported in the study). In sham stimulation condition, the double-cone coil was placed at the same location as in the real stimulation condition but was tilted by 90° to the left so that the effective magnetic field from the coil was directed away from the participant’s head. Three participants received real stimulation first and the remaining received sham stimulation first.

#### MEG Measurements

To measure sensory attenuation effect, 100 self-generated tones and 100 external tones (computer-controlled) were presented in alternating order one by one in the same sensory attenuation testing block (120 trials for each tone in each block were used for LC, who was the first participant) [55]. Both tones were 1000 Hz, 100 ms in duration and were set to be at a comfortable volume level. Tones were delivered through a plastic ear tube. The testing block started with an external tone, and then participants waited about 3 s to initiate a self-generated tone by briskly lifting their right index finger (Figure 1B). The finger lift was detected by a laser sensor which served the function of a response box. Using the laser sensor has the advantage to avoid the noise associated with the keys of a normal response box so that the only auditory input was the tone. Critically, a delay (mean = 92.0 ms; SD = 4.3 ms) was introduced between the finger lift and the tone output from the ear tube. After the self-generated tone, the next external tone was presented after a random interval between 1500 ms and 4500 ms, followed by another self-generated tone and so on until 200 tones were played. Participants received a few trials of practice before the first sensory attenuation measurement to get familiarized with the paradigm. During the practice, a ‘too fast’ visual warning signal was given if participants responded within 1500 ms from the end of the previous external tone. No warning signals were given if participants responded any time after 1500 ms. Participants were asked to close their eyes during the sensory attenuation measurement. Each sensory attenuation measurement took about 10 min, which means that it took around 25 min from the end of TMS to the end of the last sensory attenuation measurement session.

### Quantification and Statistical Analysis

#### Preprocessing

Data analysis was performed with MATLAB using FieldTrip toolbox [51] (http://www.fieldtriptoolbox.org/) conforming to recent MEG data analysis guidelines [56]. MEG signals in all testing sessions were high-pass filtered at 0.75 Hz and trials with very short inter-trial intervals (less than 1500 ms for self-generated tones) were discarded. Then very noisy trials and channels from visual inspection were rejected with ft_rejectvisual, followed by denoising using the fieldtrip function ft_denoise_pca. Rejected bad channels were repaired with interpolation methods using ft_channelrepair with default parameters. MEG signals were visually inspected again and noisy trials were discarded. Eye movement and heart artifacts were rejected using ICA (between 1 and 5 components per participant per session). After this step, 98.2 (SD = 6.8) trials and 98.7 (SD = 6.7) trials were left for self-generated tones and external tones, respectively.

#### Sensor Space Evoked Responses

MEG signals were low-pass filtered with 40 Hz cut-off frequency. Event related fields aligned to the tone onset were computed for each testing session with baseline (−600 to −200 ms) correction. In the baseline time window, time domain averaged response were compared between self-generated tones and external tones using a cluster correction to ensure that there were no significant differences in any sub-epochs [57]. The M100 component was defined in a post-stimulus time window between 70 and 100 ms and M200 component was between 125 and 155 ms. To test the existence of the M100/M200 attenuation effect for self-generated tones, paired t tests were performed on the amplitudes of evoked responses (averaging across the defined time windows) between self-generated tones and external tones. The *p* value of 0.05 was taken as the statistical significance cut-off throughout the paper. Topographies of M100 and M200 components were illustrated with the evoked responses of external tones in the pre-Real session.

#### ANOVA Analysis

For each component (M100, M200), changes of attenuation effect over testing sessions were first tested with within-subject ANOVA (implemented in Fieldtrip with ft_statfun_depsamplesFunivariate), separately for sham and real stimulation condition (Figures 3 and 5). The M100 attenuation effect was calculated by subtracting the evoked amplitude of external tones from the evoked amplitude of self-generated tones and the M200 attenuation effect was calculated the other way around by subtracting self-generated tones from external tones. This is to ensure that both M100 attenuation and M200 attenuation effects have positive values in the baseline session (due to the opposite polarity of the two evoked components). Two significant auditory sensors with the largest F values were selected for illustrating the evoked responses. To test effects associated with stimulation condition, a 2 (sham versus real stimulation) by 2 (post1 versus post2) within-subject ANOVA was performed with baseline referenced attenuation, which was obtained by subtracting the M100/M200 attenuation effect recorded during each pre-stimulation session (pre-Sham, pre-Real) from the corresponding post-stimulation sessions.

Lastly, M100 amplitude for self-generated and external tones in sham stimulation condition, where significant M100 attenuation changes were found, was subjected to a 2 (self-generated versus external) by 3 (testing sessions) within-subject ANOVA. The difference between M200 amplitude for self-generated and external tones was tested for the real stimulation condition, where significant M200 attenuation changes were found, by means of a 2 (self-generated versus external) by 3 (testing sessions) within-subject ANOVA. See results section for details.

#### Source Space Analysis

The same source localization method was used as in a previous study [4]. A semi-automatic procedure was used to co-register each participant’s T1-weighted structural magnetic resonance images to the MEG coordinate system. The initial alignment of the two coordinate systems were based on nasion and left and right pre-auricular points, which were manually identified in the individual’s structural image. Then the ICP algorithm [58] was used to achieve the numerical optimization of alignment.

The segmentation routines in FieldTrip/SPM5 were followed to create individual head models. A single shell volume conductor model [59] was used for the leadfield computation with a 10 mm grid defined on the template (MNI) brain. The template grid was then transformed into individual head space through linear spatial transformation. We used eLoreta algorithm as implemented in Fieldtrip for source space signal calculation, which was taken as the sum of the first two rank data. The covariance was calculated in the time window from −800 ms to −300 ms (relative to stimulus onset) with lambda setting at 0.07. A normalization of 0.6 was used.

For localizing evoked responses, sensor space data were processed in the same way as in the sensor space data analysis. For exploring low-frequency activities in the cerebellum, sensor space data were low-pass filtered at 10 Hz and single trial data were extracted by a matrix multiplication between sensor space data and spatial filters.

Within-subject t tests with Monte Carlo randomization (1000 permutations) were used for all source space brain-wide statistical comparisons unless otherwise specified. Multiple comparisons were corrected with false discovery rate method and only statistically significant results were reported.

M100 attenuation in the pre-Sham session was localized by comparing between the M100 component for self-generated tones and the M100 component for externally generated tones in the source space. Localization of baseline session M100 attenuation was performed similarly but with data from both pre-Sham and pre-Real sessions averaged. To find the source of the increase of M100 attenuation in the sham stimulation condition, M100 attenuation in each testing session was first calculated as the M100 source intensity difference between self-generated tones and externally generated tones through subtraction. Then the M100 attenuation in post2-Sham was compared to the average of pre-Sham and post1-Sham, as M100 attenuation only increased in the post2-Sham session.

M200 attenuation in the pre-Real session was localized by comparing between the M200 component for self-generated tones and the M200 component for externally generated tones in the source space. For baseline session M200 attenuation effect, it is compared similarly but with data from both pre-Sham and pre-Real sessions averaged. To find the source of the change of M200 attenuation in the real stimulation condition, M200 attenuation in each testing session was first calculated as the M200 source intensity difference between self-generated tones and externally generated tones through subtraction. Then the average of M200 attenuations in post1-Real and post2-Real was compared to M200 in pre-Real, as M200 attenuation started to change right after cerebellar stimulation.

To find possible pre-stimulus low-frequency changes in the cerebellum following TMS, the average amplitude of 10 Hz low-pass filtered pre-stimulus (−500 ms to 0 ms) activity was calculated for each trial in each condition. Between-subject t tests were made for the pre-stimulus activity between post1 session and pre- session, between post2 session and pre- session, which were added up to form a t-value index of pre-stimulus activity change after TMS. This t-value index was calculated separately for self-generated tones and external tones, for real and sham stimulation condition. TMS induced effect on pre-stimulus activity was investigated by comparing the t-value index between real and sham stimulation condition. Pre-stimulus activity changes over testing sessions were tested by comparing the t-value index against 0.

### Data and Software Availability

Interested readers are encouraged to contact the Lead Contact for the availability of data.

## Author Contributions

All authors designed the experiment. L.C. and D.V. performed the study. L.C. and J.G. analyzed the data. All authors wrote the manuscript.

## Supplemental Information

Document S1. Figures S1 and S2 and Table S1

Document S2. Article plus Supplemental Information
