# Supplementary material for: Role of the Cerebellum in Adaptation to Delayed Action Effects
Source: Curr Biol. 2017 Aug 21;27(16):2442–2451.e3. doi: 10.1016/j.cub.2017.06.074 (PMC5571438; doi:10.1016/j.cub.2017.06.074)
Supplement: Document S1. Figures S1 and S2 and Table S1 [file mmc1.pdf]

**Current Biology, Volume *27***

## Supplemental Information

# Role of the Cerebellum in Adaptation to Delayed Action Effects

**Liyu Cao, Domenica Veniero, Gregor Thut, and Joachim Gross**

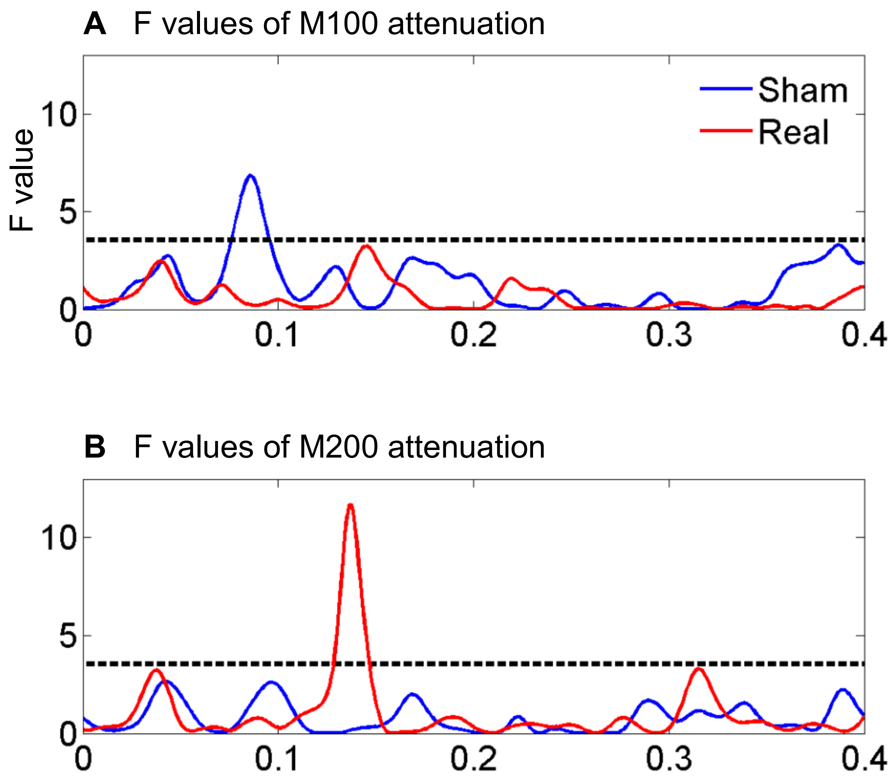


**Figure S1. ANOVA results over time for selected sensors, related to Figure 3 and Figure 5.** This ANOVA analysis is performed similarly as shown in Figure 3 and Figure 5, but this is an ANOVA performed for each time point in the time window shown. (A) There is a clear peak around 86 ms in the sham stimulation condition indicating a modulation of M100 attenuation. (B) There is a clear peak around 137 ms in the real stimulation condition indicating a modulation of M200 attenuation. The dashed line shows the uncorrected statistical significance cut-off for the ANOVA analysis.

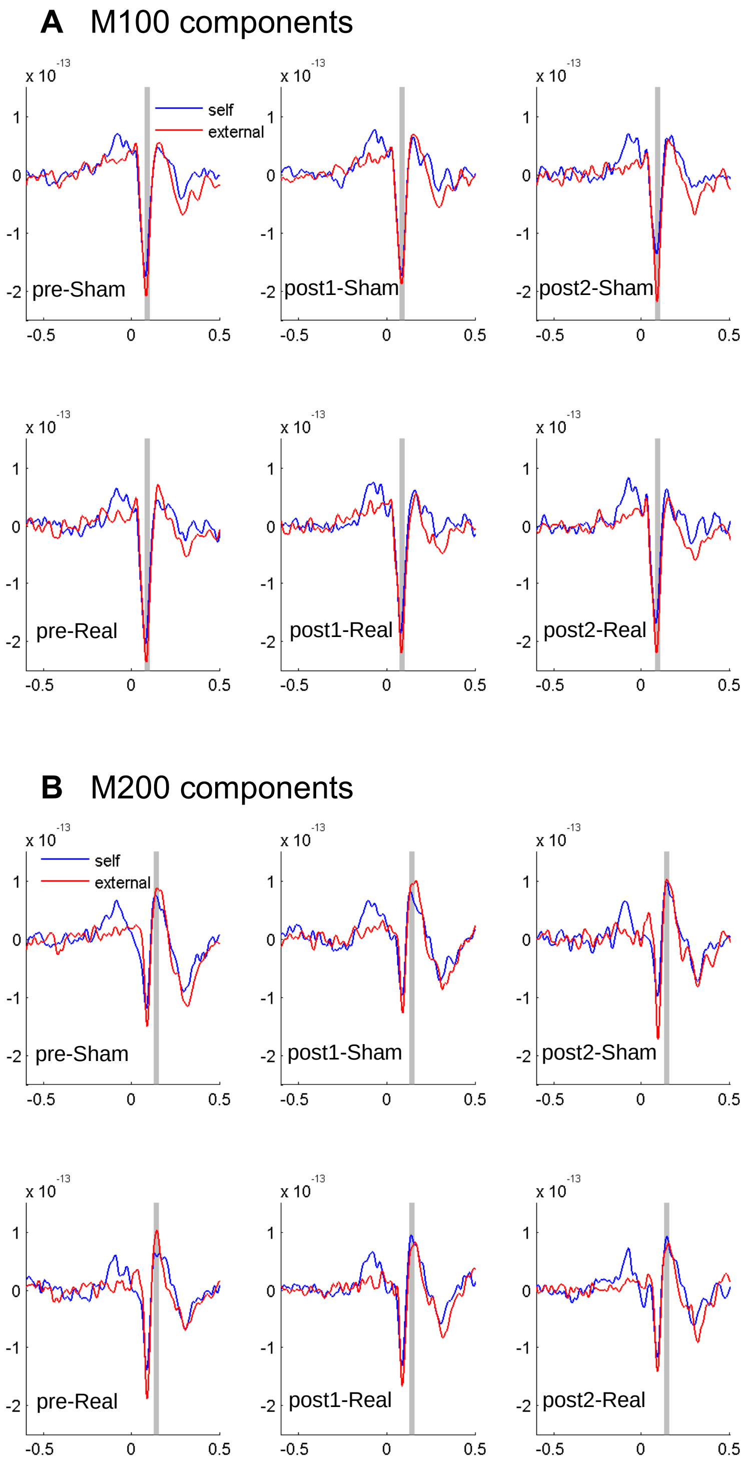


**Figure S2. Evoked components with long baseline, related to Figure 3 and Figure 5.** The figures are identical to those in Figure 3 and Figure 5 (panel A) except that the baseline window (-600 to -200 ms) is shown here. The baseline window was chosen such that it does not include the movement-related component at around -100ms. This illustrates that there are no obvious differences in the baseline window. In addition,

we statistically tested for any significant differences in the baseline time window. We performed standard cluster statistics using monte-carlo randomization. This analysis would identify time windows in the baseline that show significant differences between conditions (self-generated tones versus externally generated tones) in pre-TMS sessions for time domain averaged responses. No significant results were found.

| Testing session / Stimulation condition | pre | post 1 | post 2 |
|---|---|---|---|
| Sham | 3.26 (0.84) | 3.39 (0.75) | 3.38 (0.47) |
| Real | 3.01 (0.61) | 3.35 (0.56) | 3.46 (0.66) |

**Table S1. Mean reaction time across different conditions, related to Figure 4.** The reaction time is calculated as the interval between the end of the external tone and the onset of the next self-generated tone. Group means are show in the table (in seconds; with standard deviation in brackets). No significant interaction was found following a 2 (sham vs. real) by 3 (testing sessions: pre, post1, post2) ANOVA with the reaction time data ($F(2,18) = 0.76$, $p = 0.48$). Main effects of stimulation condition: $F(1,9) = 0.08$, $p = 0.78$; testing session: $F(2,18) = 6.43$, $p = 0.01$.
